# Effectiveness and cost-effectiveness of a transdiagnostic intervention for alcohol misuse and psychological distress in humanitarian settings: study protocol for a randomised controlled trial in Uganda

**DOI:** 10.1186/s13063-024-07980-7

**Published:** 2024-02-27

**Authors:** Catharina F. van der Boor, Dalili Taban, Wietse A. Tol, Josephine Akellot, Melissa Neuman, Helen A. Weiss, Giulia Greco, Anna Vassall, Carl May, Abhijit Nadkarni, Eugene Kinyanda, Bayard Roberts, Daniela C. Fuhr

**Affiliations:** 1 Faculty of Public Health and Policy, London School of Hygiene and Tropical Medicine, 15-17 Tavistock Place, London, WC1H 9SH UK; 2 HealthRight International, Plot 855, Mawanda Road -Kamwokya, Kampala, Uganda; 3 MRC International Statistics and Epidemiology Group, Faculty of Epidemiology and Population Health, London School of Hygiene and Tropical Medicine, Keppel Street, London, WC1E 7HT UK; 4 MRC/UVRI Uganda Research Unit, London School of Hygiene and Tropical Medicine, Plot 51-59 Nakiwogo Road, PO Box 49, Entebbe, Uganda; 5 Department of Public Health, University of Copenhagen, Bartholinsgade 4, Bg. 9, 1356 København K, CSS, Bg. 9, Building: 9.2.16, Copenhagen, Denmark; 6 Department of Prevention and Evaluation, Leibniz Institute for Prevention Research and Epidemiology, Achterstraße 30, 28359 Bremen, Germany; 7 Health Sciences, University of Bremen, Bremen, Germany; 8 Centre for Global Mental Health (CGMH), Department of Population Health, London School of Hygiene & Tropical Medicine, London, UK; 9 Addictions Research Group, Sangath, Goa India

**Keywords:** Refugees, Alcohol misuse, Mental distress, Scalable interventions, Randomised controlled trial

## Abstract

**Background:**

The war in South Sudan has displaced more than four million people, with Uganda hosting the largest number of South Sudanese refugees. Research in Uganda has shown elevated levels of alcohol misuse and psychological distress among these refugees. The World Health Organization (WHO) has developed a trans-diagnostic scalable psychological intervention called Problem Management Plus (PM +) to reduce psychological distress among populations exposed to adversities. Our study aims to evaluate the effectiveness and cost-effectiveness of the CHANGE intervention, which builds on PM + , to also address alcohol misuse through problem-solving therapy and selected behavioural strategies for dealing with alcohol use disorders. We hypothesise that the CHANGE intervention together with enhanced usual care (EUC) will be superior to EUC alone in increasing the percentage of days abstinent.

**Methods:**

A parallel-arm individually randomised controlled trial will be conducted in the Rhino Camp and Imvepi settlements in Uganda. Five hundred adult male South Sudanese refugees with (i) elevated levels of alcohol use (between 8 and 20 on the Alcohol Use Disorder Identification Test [AUDIT]); and (ii) psychological distress (> 16 on the Kessler Psychological Distress Scale) will be randomly assigned 1:1 to EUC or CHANGE and EUC. CHANGE will be delivered by lay healthcare providers over 6 weeks. Outcomes will be assessed at 3 and 12 months post-randomisation. The primary outcome is the percentage of days abstinent, measured by the timeline follow-back measure at 3 months. Secondary outcomes include percentage of days abstinent at 12 months and alcohol misuse (measured by the AUDIT), psychological distress (i.e. depression, anxiety, posttraumatic stress disorder), functional disability, perpetration of intimate partner violence, and health economic indicators at 3 and 12 months. A mixed-methods process evaluation will investigate competency, dose, fidelity, feasibility, and acceptability. Primary analyses will be intention-to-treat.

**Discussion:**

CHANGE aims to address alcohol misuse and psychological distress with male refugees in a humanitarian setting. If it is proven to be effective, it can help fill an important under-researched gap in humanitarian service delivery.

**Trial registration:**

ISRCTN ISRCTN10360385. Registered on 30 January 2023.

## Administrative information

Note: the numbers in curly brackets in this protocol refer to SPIRIT checklist item numbers. The order of the items has been modified to group similar items (see http://www.equator-network.org/reporting-guidelines/spirit-2013-statement-defining-standard-protocol-items-for-clinical-trials/).

**Table:** 

Title {1}	Effectiveness and cost-effectiveness of a transdiagnostic intervention for alcohol misuse and psychological distress in humanitarian settings: protocol for a randomised controlled trial in Uganda
Trial registration {2a and 2b}.	This study was registered with the International Standard Randomised Controlled Trial Number (ISRCTN) on the 30th of January 2023 (Ref: ISRCTN10360385). It was approved by the London School of Hygiene and Tropical Medicine ethics committee on the 3rd of March, 2023 (Ref: 28,373), and by the Mildmay Uganda Research Centre on the 27th of April, 2023 (Ref: 0401–2023).
Protocol version {3}	Version 2, 19/02/2023
Funding {4}	This study has been funded by the NIHR–Wellcome Partnership for Global Health Research (Ref: HSRP496). The funding body has had no role in the design or submission of this protocol. Furthermore, they will have no role in the collection, management, analysis, and interpretation of data.
Author details {5a}	Catharina F van der Boor^1^, Dalili Taban^2^, Wietse A. Tol^5^, Josephine Akellot^2^, Melissa Neuman^3^, Helen A Weiss^3^, Giulia Greco^1^; Anna Vassall^1^, Carl May^1^, Abhijit Nadkarni^8,9^, Eugene Kinyanda^4^, Bayard Roberts^1^, Daniela C. Fuhr1,^1,6,7^ ^1^Faculty of Public Health and Policy, London School of Hygiene and Tropical Medicine, 15–17 Tavistock Place, London, WC1H 9SH, UK ^2^HealthRight International, Plot 855, Mawanda Road -Kamwokya, Kampala, Uganda ^3^MRC International Statistics and Epidemiology Group, Faculty of Epidemiology and Population Health, London School of Hygiene and Tropical Medicine, Keppel Street, London, WC1E 7HT, UK ^4^MRC/UVRI Uganda Research Unit, London School of Hygiene and Tropical Medicine, PO Box 49, Entebbe, Plot 51–59 Nakiwogo Road, Uganda ^5^Department of Public Health, University of Copenhagen, Bartholinsgade 4, bg. 9, 1356 København K, CSS, bg. 9, Building: 9.2.16, Copenhagen, Denmark ^6^Department of Prevention and Evaluation, Leibniz Institute for Prevention Research and Epidemiology, Achterstraße 30 D-28359, Bremen, Germany ^7^Health Sciences, University of Bremen, Germany ^8^Centre for Global Mental Health (CGMH), Department of Population Health, London School of Hygiene & Tropical Medicine, UK ^9^Addictions Research Group, Sangath, Goa, India.
Name and contact information for the trial sponsor {5b}	This study is sponsored by the London School of Hygiene and Tropical Medicine (Ref: 2022-KEP-907). RGIO@lshtm.ac.uk London School of Hygiene & Tropical Medicine Keppel Street, London WC1E 7HT
Role of sponsor {5c}	The sponsor has had no role in the design or submission of this protocol. Furthermore, they will have no role in the collection, management, analysis, and interpretation of data.

## Introduction

### Background and rationale {6a}

The current civil war in South Sudan which commenced in December 2013 has caused the displacement of over four million people, of whom over two million have been displaced internationally [1]. Uganda hosts the largest number of South Sudanese refugees globally— approximately 899,000 individuals [1]. Refugees are hosted in government and United Nations High Commissioner for Refugees (UNHCR)-managed settlements [1].

It is estimated that 15–20% of South Sudanese refugees experience mild or moderate common mental health challenges including depression, anxiety, and post-traumatic stress disorder (PTSD), whilst 3–4% are estimated to have more severe mental health problems [2–4]. Furthermore, alcohol use disorders (AUD) have been identified as a significant problem amongst South Sudanese refugees, particularly amongst men who have resettled in Uganda. Although the burden is high, limited mental health and psychosocial support services are available in the settlements [5], and there are an inadequate number of specialised mental health care professionals [6, 7].

Recommendations for psychological interventions in low-resource settings include (i) focusing on task-sharing with non-specialists to deliver care, (ii) enhancing reach by targeting multiple mental health challenges simultaneously, and (iii) implementing interventions that are relevant to local cultures and contexts [8, 9]. One such evidence-based, potentially scalable, psychological intervention is the Problem Management Plus (PM +) designed by the World Health Organisation (WHO) for psychological distress in people who are exposed to adversity [10]. PM + is a transdiagnostic intervention which comprises techniques for problem solving stress management, behavioural activation, and social support. It can be delivered by trained non-specialised providers and has been proven to be effective in different settings such as Pakistan [11], Nepal [12], Turkey [13], Jordan [14] and the Netherlands [15]. A recent meta-analysis on the effects of PM + also reported evidence for the effectiveness of PM + in reducing stress indicators and promoting positive mental health [16].

Psychological distress, particularly depression and anxiety, have high comorbidity with AUD [17], and responding to both can potentially improve outcomes for both conditions. However, there are currently no evidence-based interventions that address both psychological distress and AUD. This paper provides an overview of the trial protocol to test the effectiveness of the CHANGE intervention amongst South Sudanese refugees in Uganda. CHANGE stands for “AlCohol use in HumanitariAN settings: a programme of work to address alcohol use disorders and associated adversities among conflict-affected populations in UGanda and UkrainE” [18]*.* The CHANGE intervention is a transdiagnostic intervention that addresses alcohol misuse and associated mental health co-morbidities (i.e. depression, anxiety, and posttraumatic stress symptoms). It was informed by PM + and includes additional strategies such as stress management to address alcohol misuse. These additional evidence-based strategies for addressing AUDs were identified through a meta-review [19].

### Objectives {7}

Our study aims to evaluate the effectiveness and cost-effectiveness of the CHANGE intervention in increasing the percentage of days abstinent (PDA) and reducing psychological distress; symptoms of depression, anxiety, and posttraumatic stress disorder (PTSD); disability levels; and perpetration of intimate partner violence (by drinker).

The primary hypothesis is that the CHANGE intervention together with enhanced usual care (EUC) will be superior to EUC alone in increasing PDA at the 3 months’ outcome assessment (primary outcome). The secondary hypotheses are that the CHANGE intervention and EUC will be superior to EUC alone in (i) increasing PDA at 12 months and (ii) reducing psychological distress; symptoms of depression, anxiety, and PTSD; disability levels; and perpetration of intimate partner violence at 3 and 12 months (secondary outcomes). We further hypothesise that the CHANGE intervention and EUC are cost-effective and cost-saving for the health system compared with EUC only.

### Trial design {8}

The design is a parallel-arm, superiority, single-blind, individually randomised controlled trial (RCT) with equal allocation between the two arms. A nested mixed-methods process evaluation will also be conducted to investigate competency, dose, fidelity, feasibility, and acceptability. The methodology for the process evaluation is described elsewhere [20]. This work was informed by previously completed formative research including a treatment cohort and feasibility RCT.

## Methods: participants, interventions and outcomes

### Study setting {9}

The trial will be conducted in the Rhino Camp and Imvepi settlements in northern Uganda. These settlements are largely inhabited by South Sudanese refugees who have fled the ongoing conflict between rival groups since South Sudanese independence in 2011 [21, 22]. Refugees can access free health care through 13 health posts located within the Arua district’s refugee settlements [21, 22].

### Eligibility criteria {10}

Participants will be adult (≥ 18 years) South Sudanese male refugees who meet the following eligibility criteria: (1) hazardous/harmful drinking, defined as a score between 8 and 20 on the Alcohol Use Disorder Identification Test (AUDIT)[23]; (2) having elevated levels of psychological distress defined as scoring ≥ 16 on the Kessler Psychological Distress Scale, ten-item version (K10) [24]; and (3) speaking English and/or Juba Arabic.

Exclusion criteria are: (1) men with possible alcohol dependence (AUDIT score $\ge$ 20), or non-hazardous alcohol consumption (AUDIT score < 8) [23]; (2) imminent risk of suicide/ other life-threatening risk assessed through three questions related to suicide; (3) signs of severe mental disorders such as psychosis and/or severe cognitive impairment (e.g., severe intellectual disability or dementia) assessed through a checklist of observable signs (i.e. not understanding questions, presenting with confused speech, and appearing extremely fidgety or nervous); (4) having previously received the PM + intervention; (5) having been in Uganda for less than 3 months, given that new arrivals take 2 to 3 months to relocate (i.e. receive land and refugee determination status) and that new arrivals are more likely to be in acute stages of distress in the first 3 months [25].

If, during the study, participants in the CHANGE intervention and EUC, or the EUC alone group show severe psychiatric symptoms (e.g. psychosis, suicidality), alcohol dependence, or any other symptoms that require immediate specialist treatment, they will be referred to specialist staff (e.g. psychiatric clinical officer (PCO)), which will result in exclusion from the trial. Assessors and intervention facilitators will be trained to detect this using standard operating procedures.

### Who will take informed consent? {26a}

Trained outcome assessors will carry out the informed consent process before screening and baseline. Consent will include both the screening and outcome assessments. Furthermore, a separate consent procedure will be carried out prior to the qualitative interviews. All eligible and interested participants will be invited to provide written consent. For participants who are illiterate, witnessed oral consent and a thumbprint will be requested, in line with recommendations from WHO [26]. Any adult member who is not part of the research team, and whom the participant is comfortable having present during the consent process will be eligible to provide witnessed consent.

### Additional consent provisions for collection and use of participant data and biological specimens {26b}

This trial does not involve collecting biological specimens for storage.

## Interventions

### Explanation for the choice of comparators {6b}

Participants in the intervention arm will receive the CHANGE intervention and EUC, and the control arm will receive EUC alone. EUC is the most ethical option for the control condition due to the lack of mental health services in the settlements.

### Intervention description {11a}

#### CHANGE intervention

The CHANGE intervention is a brief, transdiagnostic psychological intervention which is based on PM + and enhanced with evidence-based strategies to address problematic alcohol use. The intervention was developed through a systematic intervention adaptation process (described in a separate publication) including a meta-review of evidence-based intervention strategies [19]. PM + includes strategies such as problem-solving, stress management, behavioural activation, and accessing social support. In addition to these strategies, the CHANGE intervention includes strategies that address alcohol misuse including enhancing motivation to change behaviours and problem-solving concerns that clients face.

The CHANGE intervention will be delivered on an individual basis by a lay health care provider (“facilitator”) in six weekly sessions of around 90 min each and is divided into three phases. In the first phase (sessions 1 and 2), facilitators aim to engage the participant in the intervention, deliver psychoeducation on understanding adversity and alcohol use, identify high-risk situations and set a goal for changing the drinking behaviour together with the participant. In the second phase (sessions 2 and 3), facilitators discuss strategies on changing drinking behaviour including managing stress, emotions, and problems, and strengthening their social support. Lastly, in the third phase (sessions 4 and 5), facilitators and participants focus on relapse prevention and repeat strategies of the previous phases if required. Across the three phases, facilitators use motivational interviewing to facilitate behaviour change of patients.

#### EUC

All participants will receive EUC shortly after baseline assessment. Participants will be given a one-page information sheet detailing available resources and information on reducing alcohol intake and managing psychological distress within the settlement. This will be delivered by a member of the Village Health Team (a cadre of community health workers) who have received training on EUC.

### Criteria for discontinuing or modifying allocated interventions {11b}

During the trial, if a clinical deterioration, adverse event (AE) or serious adverse event (SAE) is detected by an outcome assessor or facilitator it will be immediately reported to the intervention team, trial sponsor, ethics committees, and DSMB. Where necessary, participants will be referred to specialist support (see section on AEs and SAEs). Clinical deterioration includes a participant being intoxicated during multiple sessions or reporting suicidal ideation. Participants who report suicidal ideation will not be referred to specialist services (or withdrawn from the trial), unless they disclose being at imminent risk of suicide (i.e. having planned and obtained the means to implement it). Participants who have entered the trial and who are subsequently referred to specialist support, either during outcome assessment or the CHANGE sessions, will be withdrawn from the trial but can still receive the CHANGE intervention.

### Strategies to improve adherence to interventions {11c}

**Fig. 1:**
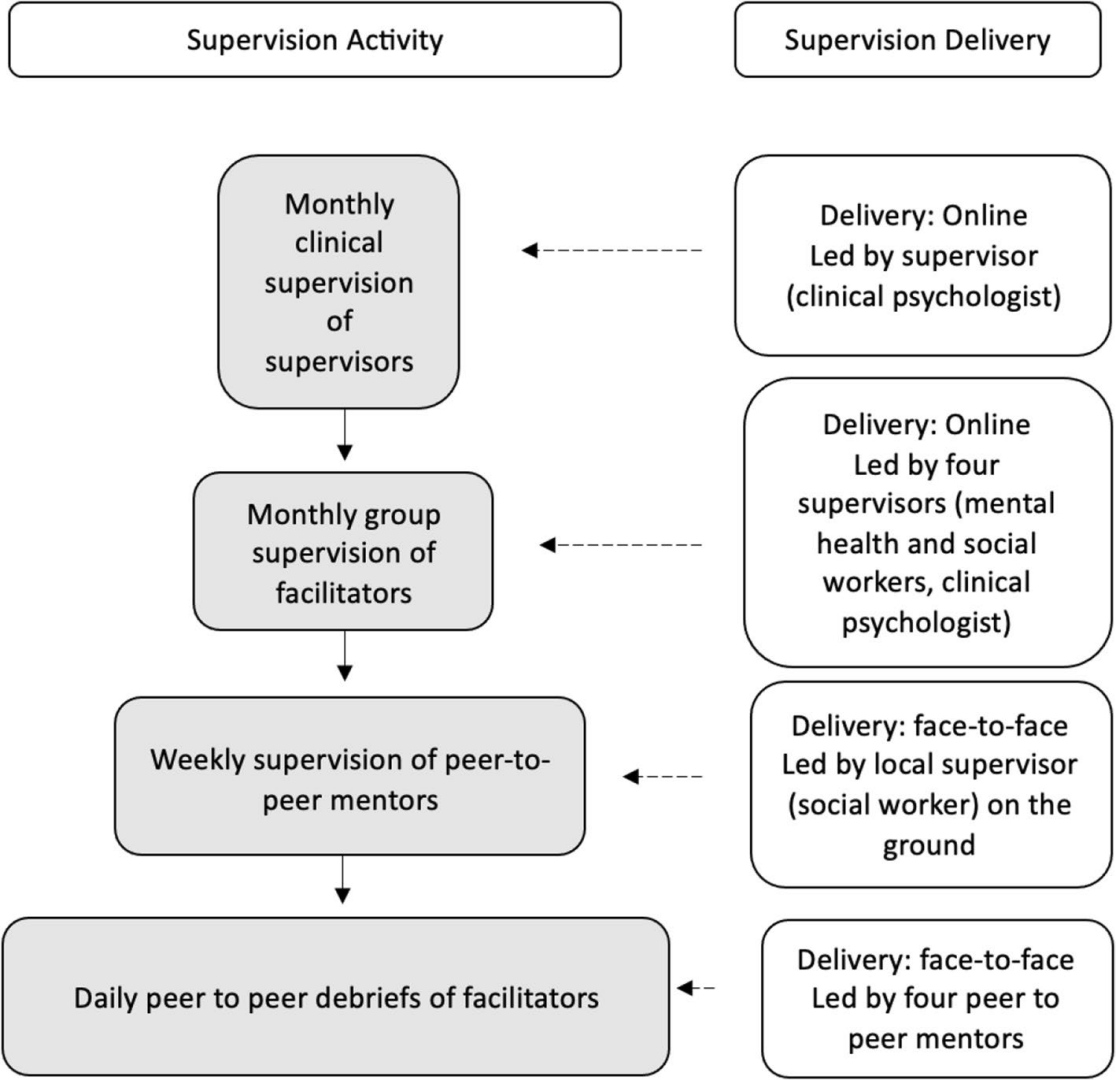
Outline of supervision during the RCT

The facilitators received an initial extensive nine-day training, followed by supervised intervention delivery to eligible participants and a 10-day refresher training with the facilitators. During the trial, ongoing supervision will be provided to the facilitators by four peer-to-peer supervisors, and four mental and/or social healthcare professionals including a clinical psychologist, two social workers and a counsellor (see Fig. 1). Supervision will include (a) daily informal peer-to-peer debrief amongst facilitators; (b) weekly group debrief with the peer-to-peer mentors and a social worker supervisor who is locally-situated; (c) monthly online group supervision with all supervisors and facilitators; and (d) monthly clinical supervision led by the clinical psychologist for the three mental and/or social healthcare professional supervisors.

Fidelity checks will be carried out in the intervention arm by audio-recording a randomly selected 10% of sessions in each phase (total *N* = 75). An external team based in Uganda which has previous experience in delivering PM + will be trained on CHANGE to carry out the fidelity assessments using a checklist of components expected to be covered in each session.

### Relevant concomitant care permitted or prohibited during the trial {11d}

Participants who have previously participated in mental health and psychosocial support interventions provided by the implementing partner, or who have received a formalised brief psychological intervention (including PM +) or alcohol-focused treatment in the previous year will be excluded from the study. Additionally, if a participant reports either being a victim or perpetrator of intimate partner violence during outcome assessment, either through the UN multicounty violence scale or during the CHANGE session delivery, he will be given support for a referral to protection services. The formal and informal support these participants will receive from protection services will be tracked throughout the trial and considered a potential moderator in the analysis of reduction in perpetration of intimate partner violence (see heading on outcomes).

Any other forms of interventions or care that participants may receive, and which are focused on physical health or wider social determinants of health (i.e. livelihood support) will be permitted.

### Provisions for posttrial care {30}

Once the trial is finished, HealthRight will continue to provide mental health and psychosocial support services for an ongoing duration within the Rhino camp and Imvepi refugee settlements.

### Outcomes {12}

Data will be collected by outcome assessors at baseline, and 3 and 12 months post-randomisation using the electronic data capturing system Open Data Kit (ODK, https://getodk.org). The outcome assessors have received a 5-day training on the administration of the assessment tools, general interviewing techniques, and ethical conduct of research. All measures will be available in English and Juba Arabic, depending on the preferred language of the participant.

**Fig. 2:**
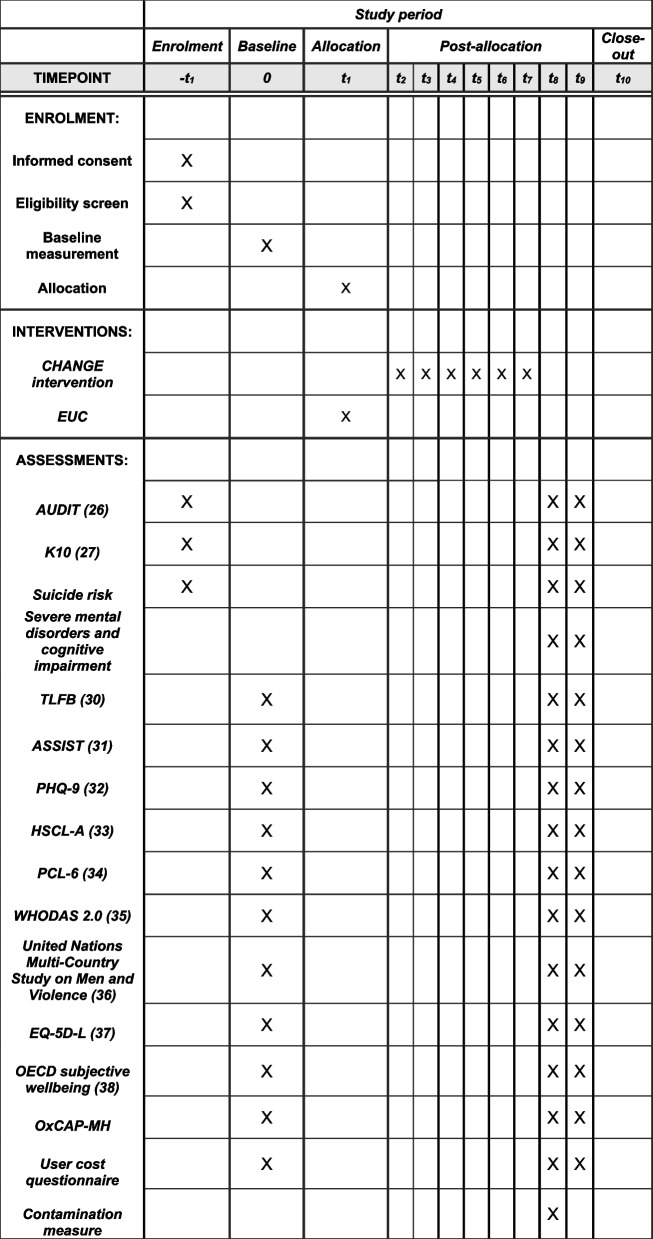
SPIRIT figure outlining the schedule of enrolment, intervention, and assessments

The primary outcome of the trial is the percentage of days abstinent in the 3-month follow-up. The secondary outcomes are the percentage of days abstinent at 12 months, a decrease in alcohol misuse, psychological distress, depression, anxiety, symptoms of PTSD, functional disability, and perpetration of intimate partner violence at 3- and 12-month follow-up. Secondary outcomes also include health economic indicators including an increase in subjective wellbeing and quality of life at 3 and 12 months follow-up [27]. The primary and secondary outcomes of the trial are further summarised in Table 1 with a more detailed description of the assessment measures in Table 2. An overview of the schedule of enrolment, intervention and assessment con be found in Fig. 2.

**Table 1:** Primary and secondary outcomes of the trial

	Source of data (see Table 2 for details of each measure)		
	Measure	End point	
		3 months	12 months
**Primary outcome**			
Percentage of days abstinent	Timeline Followback (TLFB) [28]	X	
**Secondary outcomes**			
Percentage of days abstinent	TLFB [28]		X
Alcohol misuse	• Alcohol Use Disorders Identification Test (AUDIT) [23] • Alcohol, Smoking and Substance Involvement Screening Test (ASSIST) [29]	X	X
Psychological distress	Kessler-10 (K10) [24]	X	X
Depression	Patient Health Questionnaire-9 (PHQ-9) [30]	X	X
Anxiety	Hopkins Symptom Checklist Anxiety (HSCL-A) [31]	X	X
Symptoms of PTSD	PTSD Checklist for DSM-5 (PCL-6) [32]	X	X
Functional disability	WHO Disability Assessment Schedule (WHODAS 2.0) [33]	X	X
Perpetration of intimate partner violence	United Nations Multi-Country Study on Men and Violence [34]	X	X
Health economic indicators	• EuroQol-5D-5L (EQ-5D-5L) [35] • Subjective wellbeing [36, 27] • Oxford Capabilities Mental Health questionnaire (OxCAP-MH) [37] • User cost questionnaire	X	X

**Table 2:** Details on CHANGE intervention trial outcome measures

Instrument	Description	Outcome	Contextual validity
Timeline follow-back [28]	Diary method to retrospectively assess daily estimates of drinking	Percentage days abstinent (PDA): Proportion of past 14 days on which participant was abstinent; analysis based on distribution	Validated for alcohol use assessment including in Uganda [28, 38, 39]
AUDIT [23]	Ten-item questionnaire to measure alcohol intake, identify potential dependence on alcohol; and experiences of alcohol-related harm. Each item is assessed on a scale of 0 to 4	Mean score and dichotomous as a secondary outcome	Validated with South Sudanese populations [40], and in Rhino settlement (https://www.elrha.org/project/improving-the-mental-health-of-refugee-men-through-guided-self-help-a-scalable-intervention-for-a-critical-link-in-humanitarian-programming/, Paper forthcoming)
ASSIST [29]	Eight-item screening questionnaire on estimates of alcohol use, tobacco products, and other drugs across the lifetime and in the past 3 months. Measured on a scale of 0 to 7	Mean score	Validated in a multisite international study covering Australia, Brazil, India, Thailand, UK, USA, and Zimbabwe [29]. It has also previously been validated in Rhino settlement (https://www.elrha.org/project/improving-the-mental-health-of-refugee-men-through-guided-self-help-a-scalable-intervention-for-a-critical-link-in-humanitarian-programming/, Paper forthcoming)
K10 [24]	Ten-item scale on non-specific psychological distress, scored on a scale of 1 to 5	Mean score	Validated amongst South Sudanese refugees [41], and in Rhino settlement (https://www.elrha.org/project/improving-the-mental-health-of-refugee-men-through-guided-self-help-a-scalable-intervention-for-a-critical-link-in-humanitarian-programming/)
PHQ-9 [30]	Nine-item questionnaire on prevalence of depressive symptoms assessed on a scale of 0 to 3	Mean score	Validated amongst South Sudanese refugees and in northern Uganda [42]
HSCL-A [31]	Ten-item questionnaire on prevalence of anxiety symptoms, assessed on a scale of 1 to 4	Mean score	Previously validated in multiple low and middle income settings, including Uganda [43]
PCL-6 [44]	Six-item measure on prevalence of PTSD, assessed on a scale of 1 to 5	Mean score	Good psychometric properties amongst female South Sudanese refugees in Rhino settlement [45]
WHODAS 2.0 [33]	Twelve-item measure of health and disability, which covers the domains of cognition, mobility, self-care, getting along, life activities and participation in the last 30 days. It is assessed on a scale of 1 to 4	Mean score	Good psychometric properties amongst female South Sudanese refugees in Rhino settlement [45]
United Nations Multi Country Study on Men and Violence [34]	Eleven items are used to measure the prevalence of perpetration of violence in the last 3 months. The items are assessed on a scale of 1 to 4, or 1 to 7	Prevalence of IPV split by type of intimate partner violence (physical, sexual)	Previously validated in Bangladesh, China, Cambodia, Indonesia, Sri Lanka, and Papua New Guinea [34]
EQ-5D-L [35, 46]	Five item questionnaire for measuring health related quality of life across five dimensions: mobility, self-care, usual activities, pain/discomfort, and anxiety/depression, rated on the day. A five-level response option is used	Locally adjusted scoring algorithm	Validated across different contexts including Uganda [47–49]
Subjective wellbeing [36, 27]	Five items on subjective wellbeing, measuring overall life satisfaction, whether the things the respondent does are worthwhile, and affect; scored on a scale of 1 to 5	Locally adjusted scoring algorithm	Used across countries in the Gallup world poll which covers more than 150 countries, including Uganda [36]
OxCAP-MH [37]	Sixteen-item index on wellbeing, covering various domains of individual well-being including overall health, enjoying social and recreational activities, friendship, and support, having suitable accommodation etc. Scored on a scale of 1 to 5	Mean score	Has been contextually validated by the current research team in Rhino settlement (publication forthcoming)
User Cost Questionnaire	Fifteen item measure on household expenditure specifically designed for this trial to measure the opportunity cost of participating in the CHANGE intervention	The opportunity cost of participating in the CHANGE intervention	Specifically designed and piloted for this trial. Based on the iMTA productivity loss of cost questionnaire [50]

Beyond the primary and secondary outcomes, sociodemographic data will also be collected at baseline, and a contamination measure developed for this trial will be completed at 3 months. The contamination measure will be completed by participants in both conditions and contains questions on sharing or receiving information regarding the CHANGE intervention, including specific strategies.

Competency of the facilitators was measured through the Enhancing Assessment of Common Therapeutic factors (ENACT) tool [16, 51] as part of the Ensuring Quality in Psychological Support (EQUIP) platform during their training. During the RCT, we will collect process data to investigate fidelity, dose, recruitment rates, retention/completion of follow-up and the feasibility of RCT procedures (e.g. randomisation). Furthermore, fidelity, feasibility, acceptability, and potential sustainability will be further explored in a nested qualitative study using individual semi-structured interviews with supervisors (*N* = 4), facilitators (*N* = 17), participants (*N* = 40), family members (*N* = 20) and outcome assessors (*N* = 11). The interviews will include questions on the experience of engaging with the CHANGE intervention; barriers and facilitators to attendance, delivery of the intervention; acceptability and feasibility, perceived effectiveness of the programme, helpfulness of the intervention; and opportunity costs.

### Participant timeline {13}

**Fig. 3:**
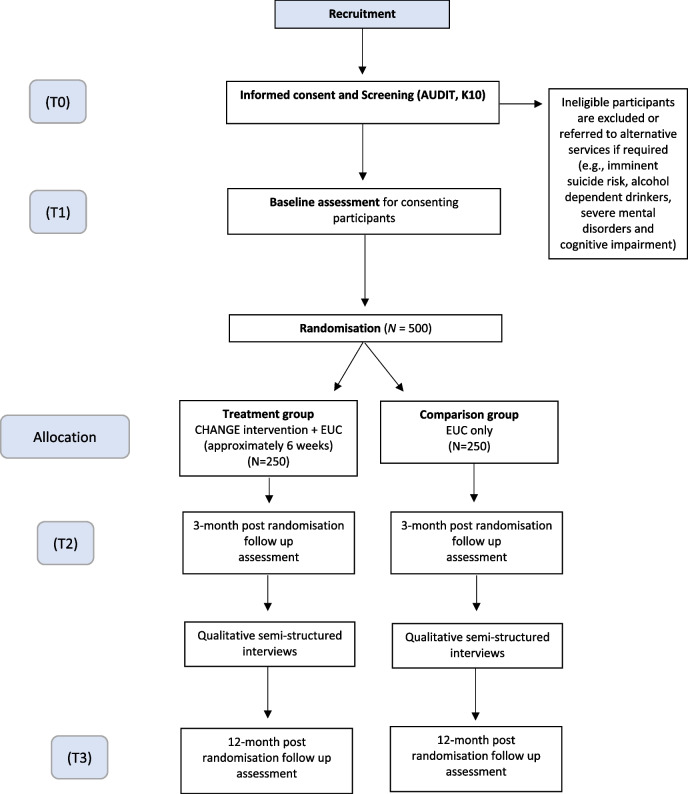
CONSORT diagram

Household recruitment will be carried out, and interested participants will be required to give informed consent prior to eligibility screening. If consent is obtained, an outcome assessor will go through sociodemographic questions, and the screening tools, as outlined in the eligibility criteria (and Fig. 3). Where inclusion criteria are met, an appointment will be made for the outcome assessor to return the next day for completion of the baseline assessment. After baseline is completed, the Village Health Team member providing EUC will carry out randomisation and deliver EUC, consisting of going through a one-page information sheet on reducing alcohol intake and managing psychological distress with the participant. Those in the intervention arm will receive their first session of CHANGE the following week. For the study flow diagram see Fig. 3.

After the 3-month follow-up, qualitative interviews will be conducted with intervention facilitators, outcome assessors, trial participants and family members to gain knowledge on implementation processes. The interviewers will be unmasked and will not be involved in outcome assessments.

### Sample size {14}

A sample size of 500 enrolled participants (250 in each arm with 1:1 allocation) will provide 90% power to detect a difference in the PDA from alcohol of 55% in the EUC arm vs 68% in the CHANGE arm (SD = 37%) at 3 months follow-up with alpha = 0.05. The sample size calculation accounts for 20% loss to follow-up at 3 months. The estimated PDA and SD are conservative, based on the CAP trial (54% vs 69%) (Nadkarni et al., 2017).

For the semi-structured interviews, all facilitators (*N* = 17), supervisors (*N* = 4), and outcome assessors (*N* = 11) will be invited to participate, plus 20 family members of intervention completers, 20 intervention completers, 20 participants in the control group, and as many participants who dropped out as possible until saturation is reached. A purposive sampling procedure will be used when selecting these participants to ensure maximum variation.

### Recruitment {15}

Rhino and Imvepi refugee settlements contain 85 villages. For our study, household recruitment will be conducted in up to 64 of the villages (excluding villages that are populated by a majority of Muslim refugees (less likely to drink alcohol due to religious reasons), refugees belonging to the Dinka ethnicity (the majority of men have returned to South Sudan), Congolese refugees (speaking different languages), or villages where the implementing partner (HealthRight) has previously recruited male participants for psychological interventions will be excluded). The order of villages to be visited will be randomised a-priori using statistical software. Residents in the villages will be screened until the required sample is reached. The date of first recruitment was the 4th of August, 2023.

## Assignment of interventions: allocations

### Sequence generation{16a}

The randomisation sequence will be generated by a statistician independent of the trial team using Stata 16.0 statistical software (College Station, TX, USA). Randomisation will be stratified by village and will be blocked in random order. Within the villages, participants will be individually randomised 1:1 to both arms of the trial after baseline assessment by the Village Health Teams.

### Concealment mechanism {16b}

Allocation concealment will be maximised using sequentially numbered opaque sealed envelopes, which will hold the randomisation code inside (generated by an independent statistician using statistical software). The envelope seal will be signed over to prevent tampering and will be opened by the Village Health Teams with the participant. Envelopes with evidence of tampering will not be used. All outcome assessors and members of Village Health Teams will be trained on the importance of maintaining the randomisation sequence and spotting tampering. Data monitoring checks will be used to identify irregularities in the sequence in which envelopes are opened.

### Implementation {16c}

Randomisation envelopes will be prepared by the unmasked data lead; each envelope will contain a randomisation code inside and an envelope ID on the outside. The envelope ID will be manually linked to the participant ID in ODK by the outcome assessor following baseline assessment. The outcome assessors will be masked to the randomisation code inside the envelope and will not know the allocation status of the participant. Once the randomisation envelope has been assigned to a participant ID, the outcome assessors will take the participant to the member of the Village Health Team and leave. The Village Health Team member will open the envelope with the participant and explain the next steps, depending on whether they are randomised into the CHANGE intervention and EUC arm, or EUC alone. The Village Health Team will deliver EUC to all participants. All study staff will be trained on the importance of maintaining the randomisation sequence, and masking, and data monitoring checks will be used to identify irregularities in the sequence in which envelopes are opened.

## Assignment of interventions: masking

### Who will be masked? {17a}

Throughout the trial, the overall PI (DF) and site PIs (WT and EK), outcome assessors and trial statisticians will be masked to treatment allocation. Participants, intervention facilitators, Village Health Team members and the project coordinator will be unmasked. The local data entry lead will also be unmasked in order to carry out routine data monitoring and cleaning.

### Procedure for unmasking if needed {17b}

There are strict standard operating procedures in place to avoid unmasking. If masking is compromised for a particular participant during outcome assessment, the assessment will stop and a new independent outcome assessor will conduct the rest of the assessment with the participant. The primary outcome will be measured early on in the survey interview to ensure it is the least likely to be compromised in the case of accidental unmasking.

## Data collection and management

### Plans for assessment and collection of outcomes {18a}

Primary and secondary outcome data, and user cost data will be collected electronically by independent outcome assessors using ODK, on tablets at baseline, 3- and 12-month follow-ups.

At baseline, additional sociodemographic data will be collected. Tablets will be password-protected, and each outcome assessor will have a specific tablet assigned to them. Data that is uploaded will be encrypted. If enrolled, participants are assigned a unique study ID that will be used to identify all participant data during data collection at the three time points.

Data checks will be conducted daily (number of records uploaded), and weekly data reports (variable summaries, summary of missing data) will be created by the data lead.

### Plans to promote participant retention and complete follow-up {18b}

Trial retention will be actively monitored at the site by the project coordinator and data lead. If participants do not attend a scheduled session or outcome assessment, a maximum of three follow-up attempts will be made by the team using both in-person and telephone methods. Contact details will be recorded for each participant including home address, a personal telephone number, as well as home addresses and telephone numbers of friends, family members, and community leaders who may be contacted (specific consent will be asked for this).

### Data management {19}

Trial data will be captured using the secure ODK server hosted by the London School of Hygiene and Tropical Medicine. Data will be collected using password-protected tablets at screening, baseline, 3 and 12-month follow-ups. A participant ID will be generated through ODK, and this ID will be used throughout the trial. The identifying key will be kept securely and separate from the rest of the data on a separate password-protected Sharepoint site that is only accessible to those who are unmasked. Data collection tablets are password protected, and data that is uploaded through ODK will be encrypted. To prevent data loss, copies of the data will be saved weekly on two password-protected external hard drives, one in Kampala and one in Arua. Standard operating procedures are in place to guide data entry, monitoring, and management, as well as a data management plan.

Data transfer procedures will be in place between the research partners to ensure anonymity, encryption, and secure storage compliance. Data will be transferred using password-protected Sharepoint. After analyses are complete (> 2 years after unmasking), data will also be made available for download in the London School of Hygiene and Tropical Medicine open data repository.

Qualitative data will be audio recorded, and transcribed. Audio recordings will be deleted from the recorders as soon as the transcription is complete. Furthermore, all identifying information will be removed from the transcripts. For both qualitative and quantitative data, no attributable data will be used in publications or presentations.

### Confidentiality {27}

The confidentiality of participants will be protected using the encrypted ODK server and unique participant ID number to identify each participant. All the members of the research and implementation team will be trained on maintaining the confidentiality of participants, including ensuring privacy when conducting interviews and assessments. Data management and security will abide by the requirements of the General Data Protection Regulations (GDPR) and any subsequent amendments.

### Plans for collection, laboratory evaluation and storage of biological specimens for genetic or molecular analysis in this trial/future use {33}

This trial does not involve collecting biological specimens for storage or evaluation.

## Statistical methods

### Statistical methods for primary and secondary outcomes {20a}

The statistical analysis plan will be finalised and approved by the Data Safety and Management Board (DSMB) prior to unmasking of the trial and analysis. Quantitative analyses will be conducted using Stata 17.0 statistical software. Initial analyses will compare baseline characteristics of study participants by arm using a descriptive analysis. If substantial baseline imbalances exist in characteristics that are likely to affect outcomes, these will be adjusted a priori in outcome analyses. The initial analysis will compare participants who did and did not consent to participate in the intervention following initial screening.

Binary outcomes will be analysed using logistic regression or negative binomial regression, depending on the distribution. Marginal standardisation and delta methods [52] will be used to calculate adjusted risk ratios if logistic regression is used. Continuous outcomes will be analysed using linear regression, after transformation if needed, and adjusted mean differences reported. Data from 3- and 12-month follow-up points will be analysed and interpreted separately.

All models will be adjusted for village using a random effect to account for village-level stratification of randomisation. Models will also be adjusted for baseline characteristics that differ substantially across arms as identified in descriptive analyses, and baseline measures of the outcome variable. Village-adjusted and fully adjusted (if appropriate) model results will be presented. All findings will be reported using CONSORT guidance [53] and intention-to-treat (ITT) principles will be used for the primary analysis of findings.

### Interim analyses {21b}

No interim analysis will be conducted. The DSMB will receive access to the unmasked data during the trial, and they will be responsible for reviewing the data and making any decisions related to the termination of the trial.

### Methods for additional analyses {20b}

For the cost-effectiveness analysis, the costs, disability-adjusted life years (DALY) averted, and quality-adjusted life years (QALY) gained will be estimated for each participant. Incremental cost-effectiveness ratios will be estimated, and uncertainty will be represented by producing a cost-effectiveness plane and cost-effectiveness acceptability curve. Furthermore, a deterministic sensitivity analysis around assumptions made on the duration of effect and discount rates will be carried out. Cost-effectiveness will be evaluated against a pre-determined threshold and compared to similar programmes addressing alcohol misuse and mental health in the region and elsewhere. Reporting of the economic evaluation will follow the recently published Consolidated Health Economic Evaluation Reporting Standards 2022 [54].

Qualitative data will be transcribed and translated into English from the language the interview was conducted in. Data will be analysed thematically using a prescheduled theoretical framework codebook drawing on Normalization Process Theory (NPT) [55] in NVivo © QSR International. The themes that come from the analysis will be mapped onto the twelve NPT constructs described in the NPT codebook [55]. The codebook will be used as a guide for interpretation of the data, rather than a scriptural authority, as recommended by May et al. [55]. Furthermore, codes, themes, and mapping onto NPT domains will be peer-reviewed in regular meetings with the wider research team to ensure the reliability of findings.

### Methods in analysis to handle protocol non-adherence and any statistical methods to handle missing data {20c}

Outcome and covariate data will be investigated for missingness, and if the missingness proportion is high (> 10%) multiple imputation methods will be considered. The outcomes will be reported using consolidated standards of reporting trials (CONSORT) guidance [56] and intention-to-treat (ITT) principles will be used for the primary analysis of findings.

### Plans to give access to the full protocol, participant-level data and statistical code {31c}

Two years after unmasking and completion of analyses, data will be stored in a publicly available repository, London School of Hygiene and Tropical Medicine Data Compass (https://datacompass.lshtm.ac.uk). The data stored will be anonymised participant data that excludes information classed as internal, confidential, or highly confidential.

## Oversight and monitoring

### Composition of the coordinating centre and trial steering committee {5d}

The current trial will be supported by a Trial Steering Committee (TSC) and a Trial Management Committee (TMC). The role of the TSC is to provide overall governance and oversight of the study and the definite RCT and ensure that it is being conducted in accordance with the protocol and the relevant regulations. The TSC provides advice to the TMC on all aspects of the trial. The TSC will meet every 6 months. Members of the TSC are experts from Uganda as well as international experts in alcohol misuse, mental health and psychosocial support, mental health in humanitarian settings and epidemiologists with expertise in conducting trials in complex settings.

The remit of the TMC is to provide country-specific advice. The members of the TMC are composed of the overall PI (DF), and the site PIs (WT and EK). The TMC will report to the DSMB and funder on the progress of the Trial.

### Composition of the data monitoring committee, its role and reporting structure {21a}

The DSMB members are independent from the trial team. The role of the DSMB is to monitor the data emerging from the RCT, as it relates to the safety of participants, and to advise the TSC on whether there are any reasons for the trials not to continue. It is the only body involved in CHANGE that has access to the unmasked comparative data during the trial. The DSMB will meet twice a year, and it will be responsible for (i) determining whether an interim analysis should be undertaken, (ii) any additional safety issues for the CHANGE project that need to be considered, (iii) report to the TSC and to recommend on the continuation of the trial, and (iii) consider any requests for release trial data and to recommend to the TSC on the advisability of this.

### Adverse event reporting and harms {22}

An overview of types of expected and unexpected SAEs and AEs in the current trial can be found in Table 3. SAEs and AEs that are reported by the participant or observed by the outcome assessors or the intervention facilitators, during any of the outcome assessment or intervention sessions will be notified to the local project coordinator and site PIs, who will be unmasked for that individual if the SAE/AE is intervention related.

**Table 3:** Expected and unexpected SAE and AEs

	Expected	Unexpected
SAE	Victimisation (violence against the trial participant or nuclear family)	Death of the trial participant due to suicide
	IPV (violence against family members by participant, or violence against participant by family member)	Death of the trial participant due to other causes
	Suicide attempt	Hospital admission of trial participant due to a psychiatric problem
	Stigmatisation	Hospital admission of trial participant due to other causes
	Serious lack of food	
AE	Clinical deterioration of the participant	
	Emotional distress caused by a trial procedure (either by the outcome assessment or the intervention delivery)	

A critical incident register will be maintained during the study to record any such events, and a specific report will be completed by the local project coordinator for each individual event. Based on the type of event, the participant will be asked to give consent for further evaluation, assessment, and/or follow-up by a designated health professional (e.g. psychologist (for stigmatisation and victimisation), PCO, or clinical officer (for all other SAEs, e.g. medical emergencies). If the participant was in the intervention arm of the trial, once they are referred to specialist care, they will not be able to re-enter the trial at a later stage. The exception to this is referrals for intimate partner violence, as participants who are referred to support services will not be excluded from the trial. Instead, they will be encouraged to access protection services and will be offered a follow-up with a social worker via the phone. Referrals for intimate partner violence will be made by an outcome assessor where the participant discloses potential intimate partner violence on the UN multi-country measure of violence during outcome assessment, or if they disclose intimate partner violence during a session with a facilitator.

Completed SAE/ AE reports, e.g. assessment by the nominated health professional will be sent to the project coordinator, who will in turn forward the same report without the participants’ ID to the site PI’s. The site PIs will compile these reports and forward to the overall PI when the reports are received. If the SAE is classified as needing immediate reporting, the site PIs will forward this report to the DSMB and the London School of Hygiene and Tropical Medicine Ethics Research Committee and/or the local IRB MildMay Uganda Research Ethics Committee (MUREC) within 3 working days of detection.

The response to SAEs related to stigmatisation will follow the same general SAE response as noted above but will also always include an evaluation by an independent PCO to ensure the quality and correct identification of these types of SAEs. For events determined by the independent clinician to be SAEs, the project coordinator will then forward the SAE for further referral to the appropriate professional. The time from initial detection of a potential SAE by the implementation team, to the project coordinator sending the SAE/AE report to the nominated healthy professional is two working days. Following these 2 days, if it classifies as an SAE, the report will be submitted to the DSMB, London School of Hygiene and Tropical Medicine ethics committee and MUREC. All SAEs and AEs will be followed up until they have abated, or until a stable situation has been reached.

### Frequency and plans for auditing trial conduct {23}

The TSC will meet twice a year to review the progress and ensure that the trial is conducted in accordance with the protocol and relevant regulations. The DSMB will also meet twice a year with the aim of monitoring the data emerging from the RCT. The DSMB is the only body involved in CHANGE that has access to the unmasked comparative data during the trial. Furthermore, a yearly report will be submitted to the Ethics Committee to detail progress. These three bodies are independent from investigators and the sponsor.

### Plans for communicating important protocol amendments to relevant parties (e.g. trial participants, ethical committees) {25}

Protocol amendments will only be implemented following approval by the London School of Hygiene and Tropical Medicine ethics committee and local MUREC approval.

### Dissemination plans {31a}

The trial has been registered in the ISRCTN public trial registry (ISRCTN10360385). The results of this study will be submitted for publication in international, peer-reviewed journals. Findings will be shared with key stakeholders (e.g. Ministry of Health, heath clusters, non-governmental organisations, community organisations) through individual country reports and briefs. Other outputs will include presentations at relevant conferences and workshops, meetings within Rhino and Imvepi refugee settlements to communicate to local stakeholders, and findings will be circulated amongst the humanitarian community on platform used by humanitarian workers (e.g. MHPSS.net and MHIN). The next steps in the research project will involve examining the scalability of the CHANGE intervention through the health system and other humanitarian sectors in Uganda.

## Discussion

The current RCT will examine the effectiveness and cost-effectiveness of the CHANGE intervention in a humanitarian context in northern Uganda. This will be one of the first rigorously evaluated interventions that aim to address alcohol misuse in a humanitarian setting. Our aim is to make the manual openly accessible if we find positive results in two RCTs (as part of the same project, we are preparing for a trial in the Ukraine).

The intervention consists of a brief, transdiagnostic, psychological intervention that targets individuals who experience psychological distress (e.g. symptoms of depression, anxiety) and AUDs. The intervention itself is delivered by trained lay health workers to South Sudanese refugees who have been affected by adversity.

The aim of the intervention is to deliver an evidence-based, potentially scalable psychological intervention that can address AUDs in conflict-affected populations. If the CHANGE intervention is proven to be effective, it will help fill an important gap in humanitarian service delivery and can be rolled out to other conflict-affected populations in different settings to test the effectiveness and cost-effectiveness of the intervention in differing contexts.

## Trial status

The current paper is based on protocol version 2 (date: 19/02/2023). The first recruitment started on the 4th of August, 2023, and the last recruitment was done on the 20th of November, 2023.

## Data Availability

The datasets generated during and/or analysed during the current study will be stored in a publicly available repository, London School of Hygiene and Tropical Medicine Data Compass (https://datacompass.lshtm.ac.uk), and will be made available for downloading one year after unblinding.

## Abbreviations

AE: Adverse event
AUD: Alcohol use disorders
AUDIT: Alcohol Use Disorder Identification Test
ASSIST: Alcohol, Smoking and Substance Involvement Screening Test
CONSORT: Consolidated Standards of Reporting Trials
DALY: Disability-adjusted life years
DSMB: Data Security and Management Board
ENACT: Enhancing Assessment of Common Therapeutic factors
EQUIP: Ensuring Quality in Psychological Support
EUC: Enhanced usual care
GDPR: General Data Protection Regulations
HSCL-A: Hopkins Symptom Checklist Anxiety
ISRCTN: International Traditional Medicine Clinical Trial Registry
ITT: Intention-to-treat
K10: Kessler Psychological Distress Scale
MUREC: Mildmay Uganda Research Ethics Committee
NPT: Normalization Process Theory
ODK: Open Data Kit
OxCAP-MH: Oxford Capabilities Mental Health questionnaire
PCL-6: Posttraumatic Stress Disorder Checklist for the DSM-5
PCO: Psychiatric clinical officer
PDA: Percentage of days abstinent
PHQ-9: Patient Health Questionnaire-9
PM +: Problem Management Plus
PTSD: Posttraumatic stress disorder
QALY: Quality-adjusted life years
RCT: Randomised controlled trial
SAE: Serious adverse event
TSC: Trial Steering Committee
TMC: Trial Management Committee
UNHCR: United Nations High Commissioner for Refugees
WHO: World Health Organisation
WHODAS: World Health Organisation Disability Assessment Schedule
