# Correction: Effectiveness and cost-effectiveness of a transdiagnostic intervention for alcohol misuse and psychological distress in humanitarian settings: study protocol for a randomised controlled trial in Uganda

**DOI:** 10.1186/s13063-024-08030-y

**Published:** 2024-03-21

**Authors:** Catharina F. van der Boor, Dalili Taban, Wietse A. Tol, Josephine Akellot, Melissa Neuman, Helen A. Weiss, Giulia Greco, Anna Vassall, Carl May, Abhijit Nadkarni, Eugene Kinyanda, Bayard Roberts, Daniela C. Fuhr

**Affiliations:** 1 Faculty of Public Health and Policy, London School of Hygiene and Tropical Medicine, 15–17 Tavistock Place, London, WC1H 9SH UK; 2 HealthRight International, Plot 855, Mawanda Road -Kamwokya, Kampala, Uganda; 3 MRC International Statistics and Epidemiology Group, Faculty of Epidemiology and Population Health, London School of Hygiene and Tropical Medicine, Keppel Street, London, WC1E 7HT UK; 4 MRC/UVRI Uganda Research Unit, London School of Hygiene and Tropical Medicine, Plot 51–59 Nakiwogo Road, PO Box 49, Entebbe, Uganda; 5 Department of Public Health, University of Copenhagen, Bartholinsgade 4, Bg. 9, 1356 København K, CSS, Bg. 9, Building: 9.2.16, Copenhagen, Denmark; 6 Department of Prevention and Evaluation, Leibniz Institute for Prevention Research and Epidemiology, Achterstraße 30, 28359 Bremen, Germany; 7 Health Sciences, University of Bremen, Bremen, Germany; 8 Centre for Global Mental Health (CGMH), Department of Population Health, London School of Hygiene & Tropical Medicine, London, UK; 9 Addictions Research Group, Sangath, Goa, India

**Correction** **:** **Trials 25, 148 (2024)**

10.1186/s13063-024-07980-7

Following publication of the original article [1], we have been notified that the Figures were out of order. These have now been rearranged.

The original article has been corrected.
